# Forensic Electrochemistry: A Dual-Mode Strategy for Rapid and Selective Detection of Catecholamine Compounds

**DOI:** 10.1021/acs.analchem.6c03301

**Published:** 2026-07-14

**Authors:** Larissa M. A. Melo, Elena Bernalte, Robert D. Crapnell, Rodrigo M. Verly, Rodrigo A. A. Muñoz, Wallans T. P. dos Santos, Craig E. Banks

**Affiliations:** † Faculty of Science and Engineering, Dalton Building, 5289 Manchester Metropolitan University, Chester Street, Manchester M1 5GD, United Kingdom; ‡ Department of Chemistry, Federal University of Vales do Jequitinhonha and Mucuri, Campus JK, Diamantina, Minas Gerais 39100000, Brazil; § Institute of Chemistry, Federal University of Uberlândia, Uberlândia, Minas Gerais 38400-902, Brazil; ∥ Department of Pharmacy, Federal University of Vales do Jequitinhonha and Mucuri, Campus JK, Diamantina, Minas Gerais 39100000, Brazil

## Abstract

A novel colorimetric–electrochemical strategy was developed for the selective detection of catecholamine compounds, with application to the analysis of epinephrine (EPN) in forensic samples as a proof-of-concept. The method integrates a colorimetric step (Melo Test), based on ferricyanide oxidation in alkaline medium (ammonium buffer), with a two-stage electrochemical protocol employing screen-printed graphite electrodes (SPE-Gr). Three analytical responses are obtained: (i) the oxidation peak of EPN before the colorimetric reaction (O_1_); (ii) a distinct reddish-orange color following a positive Melo Test for catecholamine structures; and (iii) a new oxidation signal (P_1_) corresponding to the reaction product. The combined response also enabled discrimination between β-hydroxylated catecholamines and structurally related catecholamines lacking this functionality. The method was optimized using square-wave voltammetry, demonstrating good linearity (6 to 60 μg L^–1^), low limits of detection (1.5 and 2.3 μg L^–1^ for O_1_ and P_1_, respectively), and high reproducibility (RSD <5% for *E*_p_ and *I*_p_). Application to biological matrices (urine, serum, saliva, and vitreous humor) yielded high recoveries, negligible matrix effects, and high selectivity even in the presence of structurally related compounds (18 substances individually and in mixtures). This approach offers a reliable tool for EPN screening in forensic toxicology, with potential extension to other catecholamine analytes.

## Graphical abstract

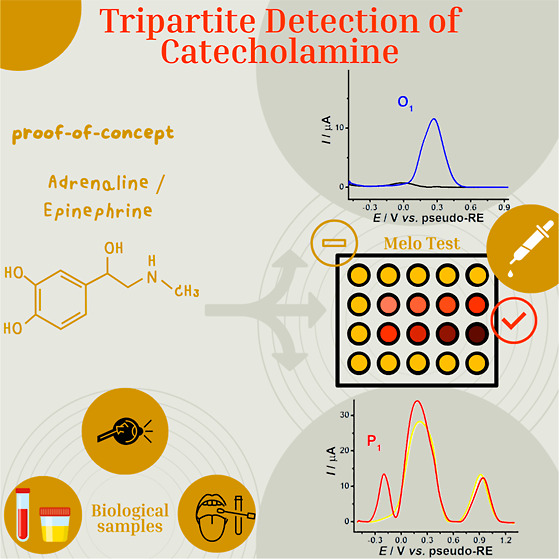

## Introduction

Catecholamines are present in a variety of bioactive compounds, including neurotransmitters such as epinephrine, endogenous metabolites, synthetic pharmaceuticals, and a range of industrial products.^1–3^ Despite their widespread occurrence, these compounds exhibit considerable toxicity and limited biodegradability, rendering them potentially hazardous to human health.^4^ These compounds contain a catechol moiety (*ortho*-dihydroxylated aromatic ring), which is highly reactive in redox transformations and coupling reactions, making catechol derivatives classical analytical targets.^5,6^

Epinephrine (EPN), a catecholaminergic compound with well-characterized physiological functions, is also of analytical interest in both forensic and clinical contexts.^7,8^ Despite its established therapeutic applications, this molecule has been implicated in cases of attempted homicide and suicide, thereby necessitating rapid and straightforward identification methods suitable for on-site screening.^9–11^ Such methods must deliver reliable results to support the timely emergency management of EPN overdose cases.^12,13^ Cases of EPN poisoning are associated with a spectrum of adverse physiological effects, including severe hypertension, arrhythmias, pulmonary edema, and myocardial ischemia.^7^ Under normal resting conditions, endogenous plasma concentrations of EPN are typically below 10 ng L^–1^, although they may increase to approximately 500 ng L^–1^ under acute stress.^14^ Therapeutic administration (e.g., intramuscular injection of 0.3 mg via autoinjector) can elevate levels to around 350 ng L^–1^. In overdose scenarios, blood concentrations may reach 1100 ng L^–1^ or higher, far exceeding physiological levels and correlating with a markedly increased risk of cardiotoxicity and fatal outcomes.^14–18^

Although numerous electrochemical and colorimetric methods have been reported for EPN detection, there is currently no standardized or officially adopted protocol for routine toxicology screening in hospital settings.^19,20^ Although classical phenol–detection reactions, such as Emerson’s test, can respond to catecholamine structures via their phenolic hydroxyl groups, they were not designed for selective recognition of catecholamines and generally exhibit poor discrimination among structurally related phenols or amine-containing metabolites.^21^ To date, no simple colorimetric method has been specifically developed for the selective identification of catecholamines while simultaneously differentiating primary and secondary amine functionalities within this class of compounds.

Electrochemical sensors, particularly those based on carbon nanomaterials, have demonstrated excellent sensitivity and selectivity, while colorimetric approaches offer simplicity and visual interpretability.^22,23^ However, each technique alone presents limitations when applied to complex biological matrices.^24,25^ The development of an integrated method that combines the rapid on-site applicability of colorimetric systems with the quantitative accuracy and molecular discrimination of electrochemical detection may offer a more reliable and selective strategy for clinical toxicology, as reported for other analytes using combined colorimetric-electrochemical approaches.^26–32^

Here, we report a colorimetric assay (termed the Melo Test) for the selective detection of catecholamine compounds using ferricyanide in ammonium buffer solution. The reaction depends on the catechol framework and generates distinct color responses according to amine substitution, with primary amines producing brown coloration, whereas secondary amines produce red coloration, consistent with the formation of conjugated quinone-imine species. When coupled with electrochemical step, the method further enables discrimination between β-hydroxylated catecholamines and structurally related catecholamines lacking this functionality. This assay was integrated with a dual electrochemical strategy providing three complementary analytical responses: (i) an initial redox signal (O_1_); (ii) a visual colorimetric response; and (iii) an oxidation signal (P_1_) attributed to the product of the positive colorimetric reaction. The method was applied to the selective determination of EPN in biologically relevant forensic matrices using a laboratory-fabricated screen-printed graphite electrode (SPE-Gr) and square-wave voltammetry (SWV). The electrochemical behavior before and after the colorimetric step, selectivity against structurally related interferents, and the proposed reaction mechanisms are discussed in detail.

## Materials and Methods

### Chemicals and Samples

The analytical standard of EPN was purchased in powder form from Sigma-Aldrich (Lancashire, United Kingdom) and dissolved in methanol to prepare a stock solution (1 mg L^–1^). This solution was employed in the colorimetric reaction (CR), combined with solutions A and B (proposed method, Melo Test), and/or diluted in a supporting electrolyte for electrochemical measurements conducted before and after the colorimetric step. Solution A was prepared by mixing 2.86 mL of ammonium hydroxide (NH_4_OH) with 0.338 g of ammonium chloride (NH_4_Cl) and diluting to a final volume of 5 mL with deionized water. Solution B was prepared by dissolving 0.16 g of potassium ferricyanide in 2 mL of deionized water. All reagents were of analytical grade and obtained from Sigma-Aldrich (Lancashire, UK). Electrochemical studies of EPN, before and after the CR, were carried out in a Britton–Robinson (BR) buffer solution (0.1 mol L^–1^), composed of an equimolar mixture of boric, phosphoric, and acetic acids. The pH was adjusted (2 to 12) using 1 mol L^–1^ NaOH. Disodium phosphate and BR buffer solutions (0.1 mol L^–1^, pH 3.0) were assessed as supporting electrolytes.

The following compounds were evaluated as potential interferents in the detection of EPN by the proposed method: glucose (GLU), fructose (FRU), uric acid (UA), citric acid (CA), ascorbic acid (AA), caffeine (CAF), melatonin, histamine, lactic acid (LA), tryptophan, glutamine, creatine, serotonin–creatinine, salbutamol, serotonin, β-estradiol, norepinephrine (NorEPN), and dopamine. All reagents were of analytical grade and were purchased from Sigma-Aldrich (Lancashire, UK). All solutions were prepared using deionized water (18.2 MΩ cm) from a Milli-Q Integral 3 system (Millipore UK, Watford, UK).

Artificial serum obtained from Sigma-Aldrich (Lancashire, United Kingdom), synthetic urine (prepared according to Laube et al.^33^), artificial saliva (prepared as described by Qian et al.^34^), and artificial vitreous humor (prepared following Thakur et al.^35^) were each spiked with 30 μg L^–1^ EPN for colorimetric-electrochemical analysis.

### Instrumental and Apparatus

Voltammetric experiments were carried out using a PGSTAT 204 potentiostat (Metrohm Autolab BV, Utrecht, The Netherlands) operated via NOVA 2.1 software. A transparent spot test was employed for the colorimetric step. The electrochemical behavior of EPN, before and after the colorimetric reaction based on the Melo test, was characterized using laboratory-fabricated screen-printed graphite electrodes (SPE-Gr), consisting of a 0.07 cm^2^ graphite working electrode, a carbon auxiliary electrode, and an Ag/AgCl reference electrode. All electrochemical measurements were performed using only the SPE pseudo-reference electrode (vs. Ag/AgCl).

### Colorimetric Step

The colorimetric reaction was carried out using light spot tests by mixing 100 μL of a 1 mg L^–1^ stock solution of EPN (positive) or methanol (blank) with 10 μL of solution A and 30 μL of solution B. A yellowish color, as observed in the negative control (methanol), indicated the absence of EPN, whereas a color shift to reddish-orange signified the presence of EPN or other catecholamine compounds. The visually perceptible color change served as a preliminary screening method for detecting EPN in biological samples of forensic interest. Electrochemical analyses were performed both before and after the colorimetric reaction. Following the reaction, a 10 μL aliquot was taken and diluted at least 10-times in BR buffer solution (0.1 mol L^–1^, pH 3) for electrochemical measurements.

### Electrochemical Measurements

Prior to each measurement, the SPE-Gr was electrochemically conditioned in a BR buffer solution at pH 3 by performing five successive scans within the potential window from −0.4 V to +0.6 V (vs. Ag/AgCl). Electrochemical investigations were carried out using cyclic voltammetry at varying scan rates and pH values. Square-wave voltammetry (SWV) and differential pulse voltammetry (DPV) were employed, with EPN detection optimized using the SWV technique on SPE-Gr. The optimal parameters were established as an amplitude of 80 mV, a step potential of 5 mV, and a frequency of 20 Hz. Voltammograms acquired via SWV were processed by background subtraction using polynomial fitting in NOVA software. Electrochemical measurements were performed both before and after the colorimetric reaction described in the Melo Test.

## Results and Discussion

### Colorimetric Reaction and Mechanistic Insights

Initially, the colorimetric test was optimized using 10 μL of solution A, 30 μL of solution B, and 100 μL of sample (methanol as the negative control and EPN as the positive control). The reagents employed in the Melo Test are designed for the selective detection of catecholamine compounds, with EPN used as a model compound in a forensic context. In contrast, for molecules containing only a single phenolic group, Emerson’s test^21^ is more appropriate for indicating its presence, the proposed mechanism for this reaction is illustrated in Scheme S1, along with key mechanistic considerations. Using the Melo Test, a yellow color was observed in the absence of EPN, while the presence of EPN resulted in an orange-red coloration. This color change was stable over time and may serve as a visual indicator of EPN. The reaction between the Melo Test reagents and catecholamine compounds was also investigated, and a mechanistic proposal is presented in Scheme 1. It can be concluded that ferrocyanide in an alkaline medium (ammonium buffer solution) act as an effective oxidizing agent, eliminating the need for the additional solution used in the Emerson’s test, 2% 4-aminoantipyrine solution (4-AAP, Solution D), to produce a visible color change.^21^

**Scheme 1:**
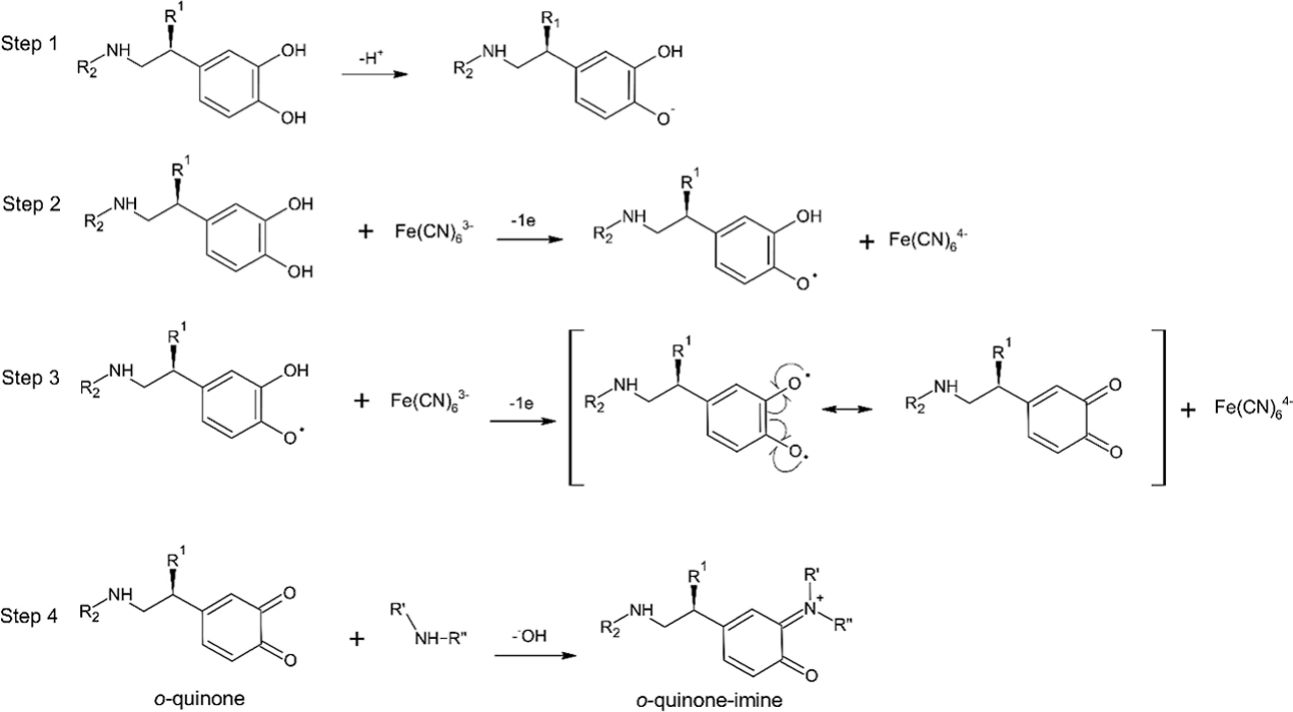
Proposed Mechanism for Catecholamine Compounds Using the Melo Test

Some conclusions regarding the mechanism can be summarized as follows:

(1) The presence of two hydroxyl groups in the *ortho* position enables direct oxidation to an *ortho*-quinone by potassium ferricyanide with high yield.
(2) *Ortho*-quinones possess extended conjugation and exhibit strong absorption in the visible region.
(3) The use of Solution D (4-AAP) is unnecessary, as the oxidation product forms aminoquinone chromophores via nucleophilic attack by intrinsic amino groups of the catecholamine substrate (e.g., epinephrine, norepinephrine, dopamine).

Aromatic imines (Ar = NR), derived from primary catecholamines, typically exhibit a brown coloration arising from extended π-conjugation involving the C=N double bond, which lowers the energy of electronic transitions and broadens light absorption. As demonstrated in Interference Studies, this color development is an intrinsic property of the catecholamine oxidation products and occurs independently of the use of Solution D. In contrast, aromatic iminium ions (Ar = NR_2_^+^), formed from substrates containing secondary amines, tend to produce a red coloration. This behavior reflects reduced effective conjugation and increased charge localization at nitrogen, which increases the transition energy and narrows the absorption profile. Additionally, subsequent reactions may occur, leading to the formation of polymeric species that contribute to the observed red coloration, as illustrated for EPN in Scheme 2. Note that tertiary catecholamines do not react under these conditions; in addition to the steric hindrance associated with a fully substituted nitrogen, nucleophilic attack on the carbonyl group cannot progress to form a C=N bond conjugated with the aromatic ring, as there is no available hydrogen on the nitrogen to enable the elimination step required for iminium formation.

**Scheme 2:**
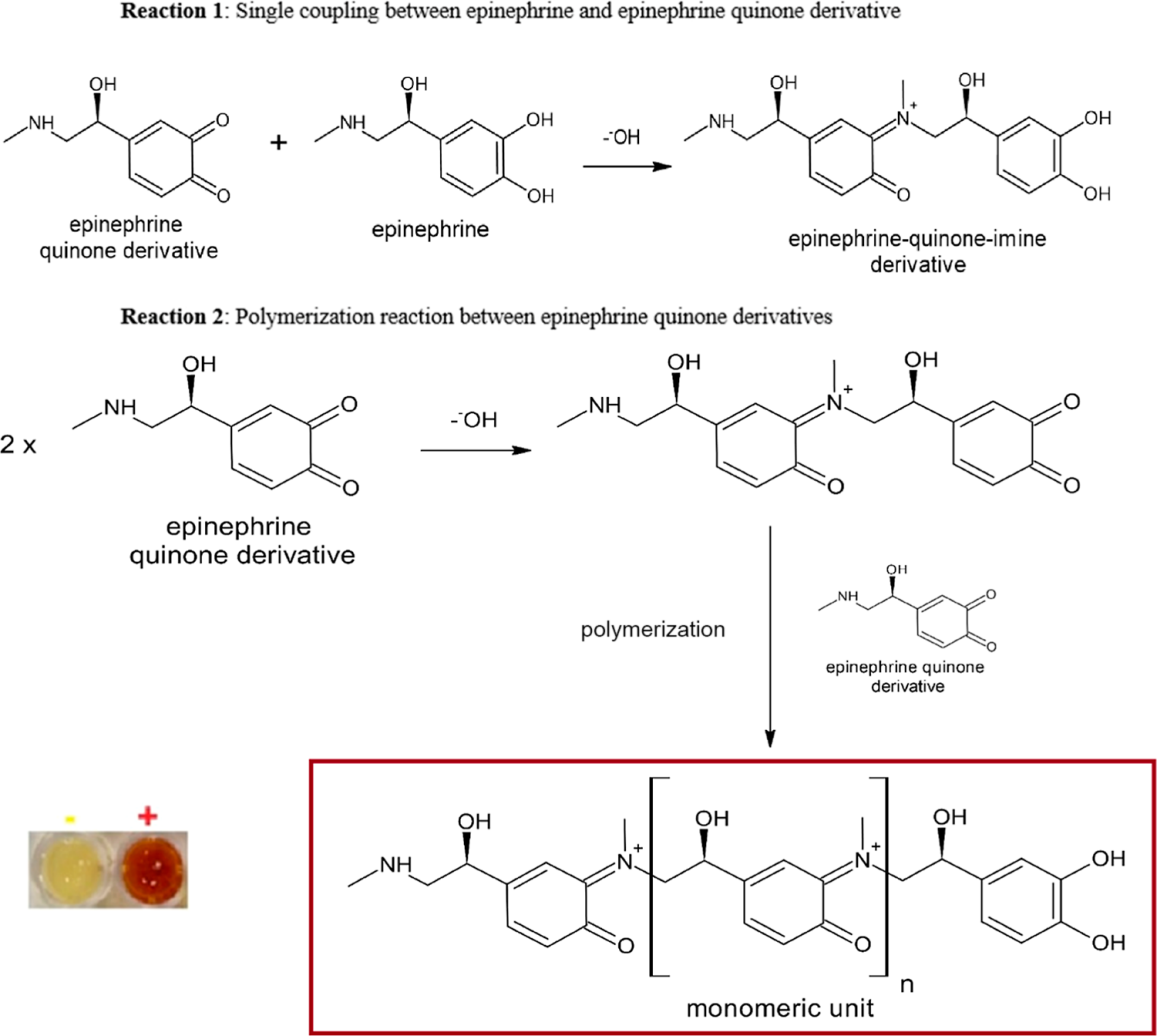
Proposed Mechanism for EPN detection Using the Melo Test

In summary, phenols bearing a single hydroxyl group require the presence of Solution D, as observed in the colorimetric test by Emerson E. (1943),^21^ to undergo further oxidation, leading to the formation of significant amounts of *para*-quinone-imine derivatives. Nevertheless, catecholamine compounds can be readily and fully oxidized by potassium ferricyanide under alkaline conditions, as demonstrated by the Melo Test, resulting in the formation of *ortho*-quinone products. These species are susceptible to nucleophilic attack by amine groups, if such functional groups are present in the substrate. As demonstrated, the Melo Test yields a positive response to catecholamine and may be employed for the detection of EPN, as well as other compounds containing two phenolic groups in their structure, including those in para-positions. In addition, electrochemical studies were systematically conducted in two stages: before and after the colorimetric reaction using the Melo Test, for a further validation of the method.

### Electrochemical Behavior of EPN before and after the Melo Test

The electrochemical behavior of EPN was subsequently evaluated in a 0.1 mol L^–1^ BR buffer solution over a pH range of 2 to 12, both before (A) and after (B) the colorimetric reaction (CR) carried out using the Melo Test, employing cyclic voltammetry (CV) on SPE-Gr, as shown in Figure S1. In the initial condition, before CR (Figure S1A), EPN displays a reversible redox couple (O_1_/R_1_), two irreversible oxidation peaks (O_2_ and O_3_), and an irreversible reduction process (R_2_) on SPE-Gr. After the CR, additional electrochemical signals corresponding to the Melo Test reagents themselves were observed, including a redox couple and an irreversible oxidation (+1.0 V vs. pseudo-RE). The P_1_ process was consistently observed across the pH range 3–12, while the P_2_ signal could be seen from pH 4 to 8 (Figure S1B and S2A).

Notably, after a positive colorimetric reaction, two new processes, designated P_1_ and P_2_, representing independent oxidation and a reduction, respectively, appeared. These signals were absent in the electrochemical profiles recorded prior to the color reaction and were not observed for the Melo Test reagents alone. Their appearance exclusively after a positive colorimetric response strongly suggests that they originate from products generated during the chemical transformation promoted by the Melo Test. This interpretation is further supported by the proposed mechanism (Schemes 2 and S2), based on the well-established electrochemistry of catechol/hydroquinone systems,^36^ the colored derivative is proposed to undergo a two-electron/two-proton oxidation to the corresponding *o*-quinone (P_1_). Under strongly acidic conditions (pH 3), the oxidized species is not efficiently reduced during the reverse scan, resulting in a single anodic process. As the pH increases, partial deprotonation of the phenolic groups facilitates electron transfer and stabilizes the quinone/hydroquinone redox couple, leading to the appearance of a quasi-reversible cathodic peak. Although definitive structural elucidation of the species responsible for P_1_ and P_2_ was not undertaken in the present work, the combined colorimetric, electrochemical, and mechanistic evidence consistently supports their assignment as markers of the positive Melo reaction.

A pH-dependent behavior of the redox processes on the SPE-Gr surface, both before and after the CR, was observed. The peak potentials (*E*_p_) of the electrochemical processes shifted toward more negative values with increasing pH, except for the O_2_ and O_3_ processes. Figure S2A presents plots of *E*_p_ versus pH for the redox events occurring on the SPE-Gr, and the corresponding linear ranges are summarized in Table S1. The slopes obtained for the pH dependence of these processes were close to the theoretical Nernstian value of 0.0592 V/pH, except for O_1_ and R_2_, which exhibited slopes near half of this theoretical value. This suggests that equal numbers of electrons and protons participate in most redox processes, whereas in the cases of O_1_ and R_2_, the number of electrons involved appears to be approximately twice the number of protons. The deviation observed in *E*_p_ versus pH behavior around pH 8–9 is attributed to the p*K*_a_ values of the EPN molecule (8.91 and 9.69).^37^ Above pH 7, the molecule exists in a mixture of protonation states, which may influence the electrochemical profile. Additionally, a correlation with peak current (*I*_p_) was observed under acidic conditions, indicating enhanced sensitivity and signal definition at low pH (Figure S2B). Based on these findings, pH 3 was selected for EPN detection, as it provided high sensitivity and well-resolved peaks for the O_1_ and P_1_ processes. Subsequently, phosphate and BR buffer solutions at the selected pH were evaluated as supporting electrolytes. The results, presented in Figure S3, revealed significant differences in peak current responses, with BR buffer providing superior analytical performance. Thus, BR was chosen as the supporting electrolyte, and the voltammetric profiles obtained under these conditions, before and after the colorimetric reaction, are displayed in Figure 1, alongside the observed color change associated with the Melo Test.

**Figure 1:**
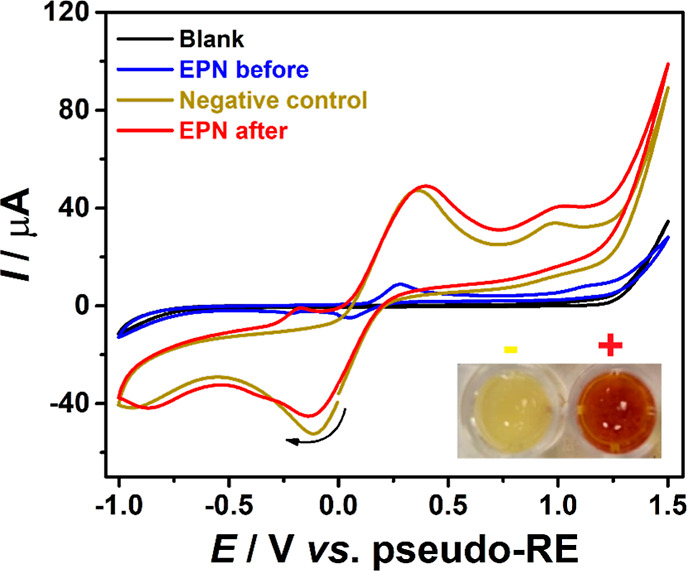
Cyclic voltammograms of 0.1 mol L^–1^ BR buffer solution pH 3 before (black line) and after addition of 66.0 μg L^–1^ EPN (blue line)—before CR; the negative control (dark yellow line) and the positive by Melo Test for presence of EPN (red line). All potential scans started at 0 V, with a scan rate of 50 mV s^–1^. Inserted the results of the Melo Test: yellow—negative control (methanol) and reddish-orange—positive for EPN presence (1 mg L^–1^).

Furthermore, to assess the dependence of the redox process signals, anodic and cathodic scans were performed, as shown in Figure S4. The redox processes of EPN before the CR were found to be electrochemically independent, Figure S4A,B. As illustrated, process P_1_ is associated with oxidation reaction that occurs after the CR, being observed exclusively when the Melo Test is positive (red voltammograms in Figure S4). It is important to highlight that P_1_ is detected only when a positive colorimetric response is observed, namely when a visible color change occurs during the reaction. This observation indicates that P_1_ is associated with products formed during the color reaction and supports its use as a secondary electrochemical marker to confirm a positive screening result. P_1_ was selected for the detection and quantification experiments (after CR) because it provided clearer signal visualization under optimized conditions than P_2_. Additionally, the mass transport behavior of the redox processes (O_1_/R_1_ and P_1_) on the SPE-Gr surface was evaluated by CV at varying scan rates (*v*) in a 0.1 mol L^–1^ BR buffer solution at pH 3 (Figures S5 and S6). The *I*_p_ for all redox processes showed a better linear correlation with the square root of the scan rate (Figures S5C and S6C) than with the scan rate itself (Figures S5B and S6B), indicating that these processes are predominantly diffusion-controlled at the SPE-Gr surface. Furthermore, the logarithmic plots of *I*_p_ versus *v* yielded linear relationships (Figures S5D and S6D), with the corresponding regression equations summarized in Table S2. The slopes obtained (≤0.5) corroborate diffusion-controlled charge transport for the evaluated processes.

### Electroanalytical Performance by SWV

The electroanalytical performance of EPN, before and after the CR using Melo Test, was subsequently investigated by SWV. The optimal conditions were established using an amplitude of 80 mV, a step potential of 5 mV, and a frequency of 20 Hz. Under these parameters, repeatability (intraelectrode, *N* = 5) and reproducibility (interelectrode, *N* = 3) studies were first carried out for the determination of EPN on the SPE-Gr (Figure 2).

**Figure 2:**
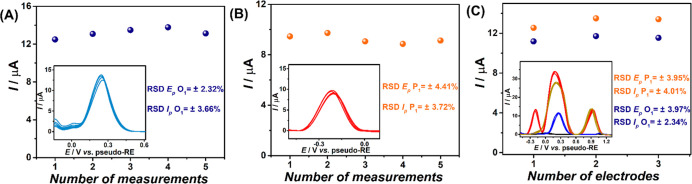
Plot of *I*_p_ vs number of measurements performed on the same SPE-Gr (intraelectrode) (A and B) and on three distinct SPEs (interelectrodes) (C). Insets are voltammograms recorded by SWV of 30.0 μg L^–1^ EPN in 0.1 mol L^–1^ BR buffer solution at pH 3 before CR—blue line (A and C) and after CR—red line (B and C). (C) Insets are SWVs of 0.1 mol L^–1^ BR buffer solution pH 3 before (black line) and after addition of 30.0 μg L^–1^ EPN (blue line)—before CR; the negative control (dark yellow line) and the positive by Melo Test for presence of EPN (red line), for three distinct SPEs. Experimental conditions: amplitude of 80 mV, step potential 5 mV, and frequency 20 Hz. In dark blue O_1_ and in dark orange P_1_ data.

These results demonstrated good stability in the electrochemical responses of EPN, both before and after the CR, with low relative standard deviations (RSDs) for *E*_p_ and *I*_p_ (<5%) across measurements performed on the same electrode and on three different electrodes. It is important to note that measurements on the three distinct electrodes were performed using three independently prepared solutions, as shown in Figure S7, where the colorimetric step also exhibited high reproducibility, with visually consistent color responses for both negative and positive (EPN presence) samples, therefore, confirming the stability of the Melo Test. As shown in Figure 2, the *E*_p_ values for O_1_ and P_1_ remained consistent across all measurements (RSDs <4.5%), highlighting the suitability of this method as a screening approach for identifying EPN before and after the CR.

Next, the optimal linear range for the colorimetric determination of EPN using the Melo Test was evaluated between 60 and 660 μg L^–1^, whereas the electrochemical detection using SWV applied both before and after the CR, was assessed using 10-fold more diluted solutions (6–66 μg L^–1^), as depicted in Figure 3.

**Figure 3:**
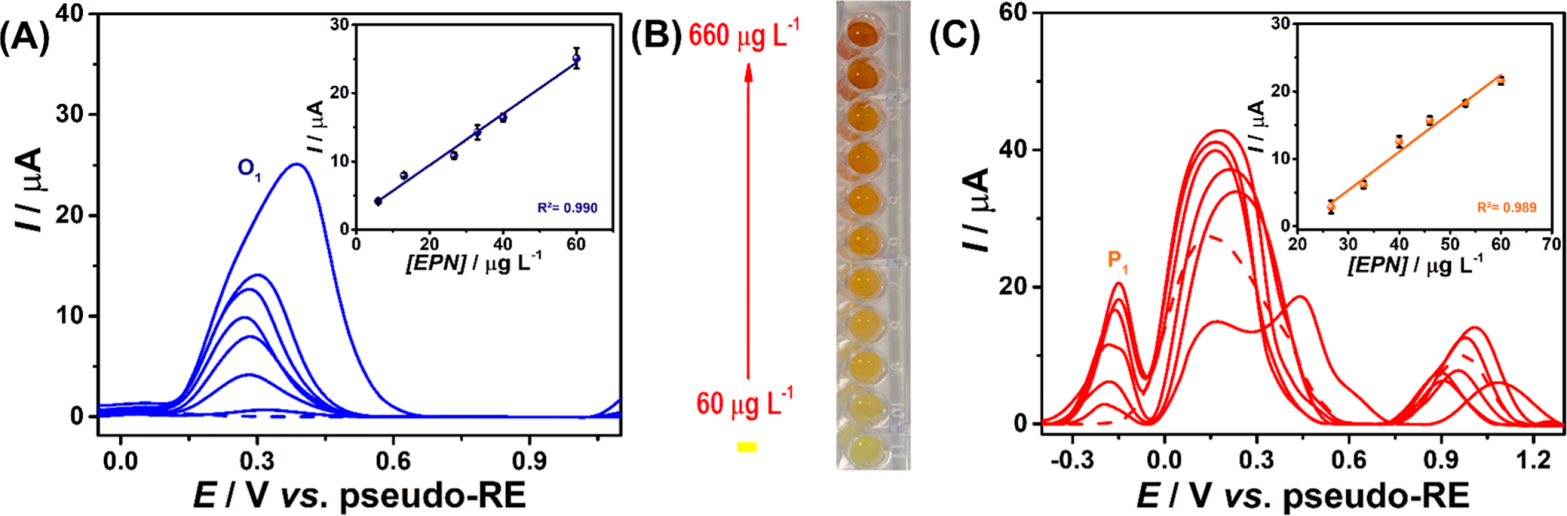
(A) Square-wave voltammograms in 0.1 mol L^–1^ BR buffer solution (pH 3) on SPE-Gr before (dashed line) and after (solid line) addition of 6–66 μg L^–1^ EPN. Inset show linear regression for O_1_—all measurements were performed in triplicate, and the error bars (black) were smaller than the symbol (dark blue) for *I*_p_. (B) Results of the Melo Test for negative control (symbol “-” yellow) and solutions in concentrations 60–660 μg L^–1^ EPN. (C) Square-wave voltammograms of these solutions diluted × 10 in 0.1 mol L^–1^ BR buffer solution (pH 3). Inset show linear regression for P_1_—all measurements were performed in triplicate, and the error bars (black) were smaller than the symbol (dark orange) for *I*_p_. The experimental conditions were the same as in Figure 3.

As shown in Figure 3A, good linearity (*R* ^2^ = 0.99) was obtained in the range of 6–60 μg L^–1^ for EPN before the CR by O_1_ (inset, Figure 3A). A visible color change compared to the negative control in the Melo Test was observed at EPN concentrations from 200 μg L^–1^ (Figure 3B). For the electrochemical detection of EPN after the CR, based on the P_1_ signal, good linearity (*R* ^2^ = 0.99) was achieved in the range of 26.6–60 μg L^–1^ (inset, Figure 3C). The linear regression equations obtained for the O_1_ and P_1_ processes are summarized in Table S3. The limit of detection (LoD) for the O_1_ and P_1_ processes was determined to be 2.3 μg L^–1^ and 1.5 μg L^–1^, respectively, using the equation 3*S*_B_/*m*,^38^ where *S*_B_ is the standard deviation (*N* = 10) of the blank response and *m* is the slope of the corresponding calibration curve. The limits of quantification (LoQ) for O_1_ and P_1_ were calculated as 7.4 μg L^–1^ and 5.0 μg L^–1^, respectively, based on the equation 10*S*_B_/*m*.^38^ It is worth noting that although EPN concentrations in biological fluids in cases of intoxication or fatal outcomes are rarely standardized, values between 10 and 1000 μg L^–1^ have been reported in blood or serum, and 50 to 5000 μg L^–1^ in urine and 1 to 50 μg L^–1^ in vitreous humor, according to forensic case studies described in the literature.^39–42^ The detection levels achieved for EPN are sufficiently low to permit application to clinical and forensic samples associated with poisoning cases within the concentration ranges reported in the literature. Accordingly, the proposed method offers suitable sensitivity for EPN screening by integrating Melo test with electrochemical measurements performed before and after the CR, enabling both selective identification and quantitative determination of the analyte. Therefore, the O_1_ and P_1_ peaks may be employed as electrochemical indicators of the presence of EPN in the samples, before and after the CR, respectively. Accordingly, the proposed method delivers three distinct analytical responses: (1) the electrochemical behavior of EPN prior to the CR (O_1_), (2) the visual color change observed in the Melo Test, and (3) the electrochemical behavior of EPN following the CR (P_1_). This robust approach enables the on-site identification of EPN in forensic samples contributing to real screening analyses.

### Interference Studies

Interference studies were conducted to evaluate the influence of potentially coexisting compounds on the reliable identification of EPN, which is critical for assessing the selectivity of the proposed combined methodology and viability for real applications. First, electroanalytical detection of EPN, before and after the CR, were evaluated in the presence of antioxidant species and endogenous compounds commonly found in biological matrices (GLU, FRU, UA, CA, AA, and CAF). The results obtained are included in Figure S8.

Figure S8A shows that UA and AA exhibited oxidation processes at +0.34 V and +0.22 V (vs. pseudo-RE), respectively, under the experimental conditions applied before the CR. The *E*_p_ of these species are very close to that of EPN (+0.33 V vs. pseudo-RE), potentially resulting in signal overlap and hindering selective identification by electrochemical means alone. In contrast, Figure S8B demonstrates that the colorimetric step is highly selective for EPN when compared with FRU, GLU, UA, CAF, AA, and AC, as only the presence of EPN produced a visible color change (reddish-orange) relative to the negative control (yellow). Additionally, Figure S8C shows that the P_1_ process was observed exclusively for EPN, when the CR was positive, further confirming the selectivity of the proposed method when combining both electrochemical and colorimetric for EPN identification.

Second, the developed methodology was applied to determine EPN in the presence of its own metabolites (melatonin, histamine, LA, tryptophan, glutamine, and creatine) as included in Figure S9. In Figure S9A, it can be observed that only melatonin and tryptophan exhibited electrochemical responses with *E*_p_ sufficiently distinct from that of O_1_, minimizing the likelihood of signal overlap. Similarly, the results from both the colorimetric and electrochemical steps following the CR demonstrated high selectivity toward EPN, with a visible color change and the appearance of the P_1_ signal occurring exclusively in the presence of EPN. The colorimetric results (Figures S8B and S9B) are supported by the chemical structures (Figures S8D and S9D) of the analyzed molecules, which do not contain EPN-like reactive sites (−OH ×2).

Third, structurally related catecholamines and other biologically relevant compounds (serotonin–creatinine, salbutamol, serotonin, β-estradiol, nor-EPN, and dopamine) were investigated (Figure 4). All compounds evaluated exhibited oxidation processes under the proposed conditions. Nor-EPN, dopamine, serotonin, and serotonin–creatinine showed oxidation peaks at +0.35 V, +0.29 V, +0.36 V, and +0.30 V (vs. pseudo-RE), respectively. These values are close to the O_1_ peak of EPN (+0.33 V vs. pseudo-RE), which is consistent with oxidation of structurally related electroactive groups and, particularly for catecholamines, reflects the common catechol moiety. Furthermore, nor-EPN and dopamine also produced positive responses in the Melo Test, yielding brown coloration clearly distinct from the reddish-orange response observed for EPN (Figure 4D). This behavior was expected, as both compounds contain catechol and primary amine functionalities capable of participating in the proposed coupling reaction. The different color response, however, indicates that amine substitution plays a decisive role in product formation, in agreement with the mechanism discussed in Colorimetric Reaction and Mechanistic Insights. Although dopamine displayed an initial oxidation response similar to that of EPN, the secondary electrochemical signal associated with the color-reaction product was markedly reduced or even undetectable. This finding is consistent with the absence of the β-hydroxylated side chain in EPN and nor-EPN-type adrenergic structures, suggesting that the second analytical response is sensitive to structural differences beyond simple catechol oxidation. Accordingly, the combined colorimetric-electrochemical approach enables discrimination between β-hydroxylated catecholamines and less substituted catecholamine analogues.

**Figure 4:**
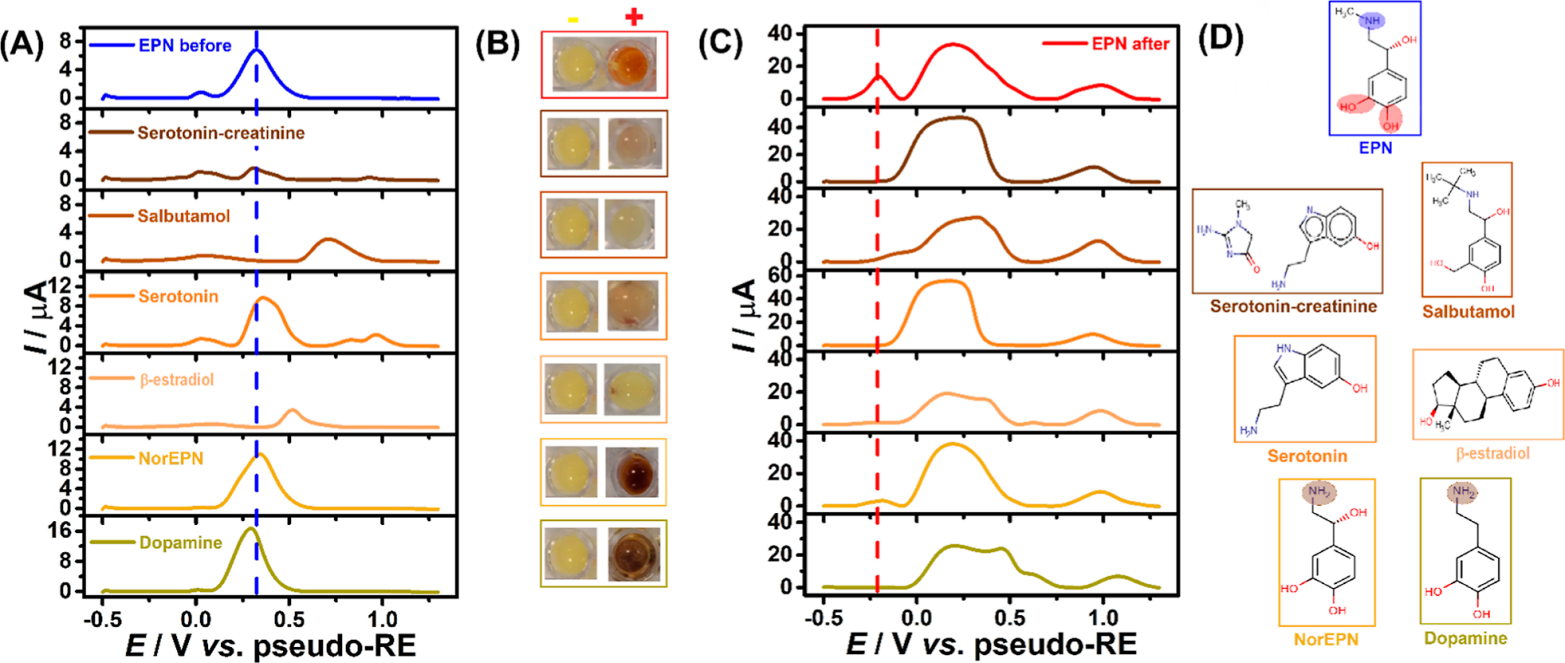
Square wave voltammograms before (A) and after (C) CR on SPE-Gr. EPN before (blue line) and after (red line) Melo Test. All compounds were at 30.0 μg L^–1^ in 0.1 mol L^–1^ BR buffer solution at pH 3. The experimental conditions were the same as in Figure 3. (B) Results of the Melo Test: (−) negative control and (+) EPN, Serotonin-creatinine, Salbutamol, Serotonin, β-estradiol, nor-EPN and Dopamine, all at 0.6 mg mL^–1^. (D) Chemical structure of all analytes in the same colors as (A).

This discrimination is unlikely to originate from the initial electrochemical oxidation, as catecholamines containing the catechol moiety are oxidized at similar potentials. Instead, the selectivity most likely arises during the subsequent chemical reaction, in which the electrogenerated *o*-quinone reacts with the nucleophilic reagent. The β-hydroxyl substituent of EPN plays an important role by influencing the electronic distribution and stereochemical arrangement of the side chain, promoting polymerization into extended chains that generate the characteristic reddish-orange chromophore. Although Nor-EPN also contains a β-hydroxyl group, its primary amine, rather than the *N*-methyl secondary amine present in EPN, alters the coupling reaction, resulting in the observed brown coloration and a distinct electrochemical profile. These observations are consistent with previous reports by Goodwin et al. (2006), which demonstrated that subtle structural variations adjacent to the aminoethyl side chain significantly influence quinone-derived coupling reactions and the nature of the resulting colored products.^36^ In contrast, compounds lacking the β-hydroxyl group, such as dopamine, or containing a primary amine instead of the *N*-methyl secondary amine present in EPN, exhibit different electronic and steric environments, leading to the formation of different chromophores and electrochemical responses following the Melo reaction.

Finally, to assess performance under more complex conditions, all compounds were also analyzed in binary mixtures containing EPN, as illustrated in Figure S10. The O_1_ and P_1_ redox processes, together with the expected reddish-orange color development, were consistently observed in most mixtures containing EPN (Figure S10), except those containing serotonin or nor-EPN. In the first case, the serotonin mixture exhibited an atypical profile, characterized by suppression of the P_1_ signal (Figure S10C), suggesting possible competitive reactions or inhibition of the secondary oxidation pathway. In contrast, the mixture containing nor-EPN produced a brown color response in the Melo Test (Figure S10B), reflecting the competing contribution of a structurally similar primary amine catecholamine.

Recovery data obtained at a 2:1 EPN-to-interferent ratio (Figure S11) showed near-quantitative O_1_ recoveries for most compounds, except for the serotonin–creatinine mixture, where substantial signal attenuation (∼50%) was observed. In comparison, the P_1_ process remained stable in the presence of several interferents, with recoveries close to 100% for β-estradiol, dopamine, and serotonin–creatinine. However, salbutamol and nor-EPN showed increased recoveries (∼150%), indicating a possible additional contribution to the voltammetric P_1_ signal. Overall, although partial overlap in the primary oxidation process was observed for structurally related compounds, the combined interpretation of color pattern, O_1_ response, and P_1_ behavior allowed effective discrimination of EPN, supporting the selectivity of the dual-response method.

### Application in Forensic Samples

Four biological samples of forensic interest (artificial vitreous humor, synthetic urine, artificial saliva, and serum) were spiked and evaluated using the proposed methodology for the identification of EPN (Figure S12). All samples were spiked with 0.6 mg mL^–1^ of EPN for the colorimetric test and subsequently diluted to 30 μg L^–1^ in BR buffer solution (pH 3) for electrochemical analysis. The presence of low concentrations of EPN in the different matrices was confirmed through the characteristic color change (Melo Test, inserted Figure S12) and the electrochemical signals observed before and after the colorimetric reaction (O_1_ and P_1_). As shown in Figure S12, both the colorimetric and electrochemical responses in all matrices closely matched those obtained for the EPN standard, supporting the applicability and reliability of the proposed strategy for forensic sample analysis.

Furthermore, addition–recovery experiments were conducted using the selected matrices to assess potential matrix effects on the detection and quantification of EPN. Recovery values of 94.8 (±2.8)%, 106.1 (±4.2)%, 97.7 (±2.9)%, and 93.9 (±3.8)% using O_1_ and 107.2 (±3.2)%, 109.3 (±4.3)%, 112.1 (±4.5)%, and 87.9 (±3.5)% using P_1_ were obtained for vitreous humor, saliva, serum, and urine, respectively. In all matrices evaluated, EPN could be quantified with recoveries close to 100%, indicating no significant matrix effects. Although the present study was conducted using spiked matrices, authentic forensic samples may present additional challenges, including variable analyte stability, increased matrix complexity, and the potential need for sample pretreatment. It is important to note that within the current forensic context, the primary objective of screening tests is the selective identification of target substances rather than their quantification. Nonetheless, the proposed method represents a promising alternative for on-site application as a rapid, simple, and selective screening tool for both the identification and quantification of EPN in forensic samples.

It is worth noting that UV–vis spectrophotometric methods for the determination of EPN have previously been reported, demonstrating satisfactory analytical performance in pharmaceutical and biological samples.^43,44^ Similarly, the Emerson reaction has been successfully combined with UV–vis measurements for the detection of bendiocarb and other analytical applications.^45–47^ For example, Fiamegos et al. (2000) reported the determination of phenols compound using the Emerson’s reaction with 4-AAP, achieving a limit of detection of 3 μg L^–1^ and a linear range of 5–400 μg L^–1^.^47^ These approaches rely exclusively on absorbance measurements as the sole analytical response and may be more susceptible to matrix interferences when applied to complex samples, such as seized forensic materials and toxicological specimens.

In contrast, the strategy proposed here in combines the Melo colorimetric reaction with electrochemical interrogation of the same solution medium before and after the colorimetric step, providing three complementary analytical responses. In addition to the visible color change, the appearance of the P_1_ electrochemical signal exclusively following a positive Melo Test response provides an additional analytical marker that is absent in the original EPN solution and reagent blanks. This complementary information enhances the reliability of analyte identification and may be particularly advantageous for the forensic screening of colored or compositionally complex samples, in which conventional UV–vis measurements can be affected by matrix interferences. Furthermore, the portability and low cost of the proposed electrochemical platform, compared with conventional UV–vis instrumentation, supports its application in decentralized laboratory settings and facilitates rapid access to analytical information during forensic investigations.

Unlike conventional colorimetric or electrochemical methods used independently, the proposed dual-mode platform provides three complementary analytical responses from the same sample. The initial electrochemical response (O_1_) enables the detection of EPN before the CR, while the Melo Test provides a rapid visual indication of the presence of a reactive catecholamine. Subsequently, the appearance of the P_1_ signal following a positive CR provides an additional confirmation step. This multiresponse strategy enhances selectivity and analytical reliability by reducing the likelihood of false-positive results arising from matrix interferences or structurally related compounds. These characteristics are particularly relevant for forensic screening applications, where rapid, portable, and highly selective identification tools are required.

## Conclusions

We report on the development of a novel colorimetric assay (Melo Test) for the selective detection of compounds containing catechol moieties. When coupled with a dual-stage electrochemical protocol, the method produced three distinct and complementary analytical responses. The combined platform enabled reliable detection of EPN through: (i) the initial redox signal (O_1_); (ii) a characteristic reddish-orange color response following a positive Melo Test; and (iii) a second electrochemical signal (P_1_) arising from the reaction product. Importantly, the integration of colorimetric and electrochemical measurements provided a level of selectivity and reliability that could not be achieved by either technique alone. The requirement for agreement between the O_1_ signal, the characteristic color response, and the P_1_ signal establishes a multiparameter identification strategy that is particularly advantageous for forensic screening of complex samples. The dual-responses also enabled discrimination between β-hydroxylated catecholamines and structurally related catecholamines lacking this functionality. The method was validated in biological matrices of forensic relevance, demonstrating consistent performance with relative standard deviations (RSD) below 5% for both *I*_p_ and *E*_p_, low limits of detection (2.3 μg L^–1^ and 1.5 μg L^–1^) and quantification (7.5 μg L^–1^ and 5.0 μg L^–1^), and effective tolerance to matrix effects. These results support the application of this platform as a practical and selective tool for EPN screening in forensic toxicology, with potential applicability to other catecholamine analytes.

## Supplementary Material



## Notes

Proposed mechanism, cyclic voltammograms, plots of Ep and I_p_ vs pH, table of linear relationships, plots of peak current against scan rates, table of linear regression equations, square-wave voltammograms and recovery results (PDF)
