# Supplementary material for: Forensic Electrochemistry: A Dual-Mode Strategy for Rapid and Selective Detection of Catecholamine Compounds
Source: Anal Chem. 2026 Jul 14;98(29):21860–9. doi: 10.1021/acs.analchem.6c03301 (PMC13425554; doi:10.1021/acs.analchem.6c03301)
Supplement: Supplementary file 1 [file ac6c03301_si_001.pdf]

Supporting information

Forensic Electrochemistry: A Dual-Mode Strategy for Rapid and Selective Detection of Catecholamine Compounds

Larissa M. A. Melo[a,b], Elena Bernalte[a], Robert D. Crapnell[a], Rodrigo M. Verly[b], Rodrigo A. A. Muñoz[c], Wallans T. P. dos Santos[*d] and Craig E. Banks[*a]

[a] *Faculty of Science and Engineering, Manchester Metropolitan University, Dalton Building, Chester Street, M1 5GD, Great Britain*

[b] *Department of Chemistry, Federal University of Vales do Jequitinhonha and Mucuri, Campus JK, 39100000, Diamantina, Minas Gerais, Brazil*

[c] *Institute of Chemistry. Federal University of Uberlândia, 38400-902 Uberlândia, Minas Gerais, Brazil*

[d] *Department of Pharmacy, Federal University of Vales do Jequitinhonha and Mucuri, Campus JK, 39100000, Diamantina, Minas Gerais, Brazil*

[*]To whom correspondence should be addressed.

E-mail: wallanst@ufvjm.edu.br and c.banks@mmu.ac.uk; Tel: +44(0)1612471196

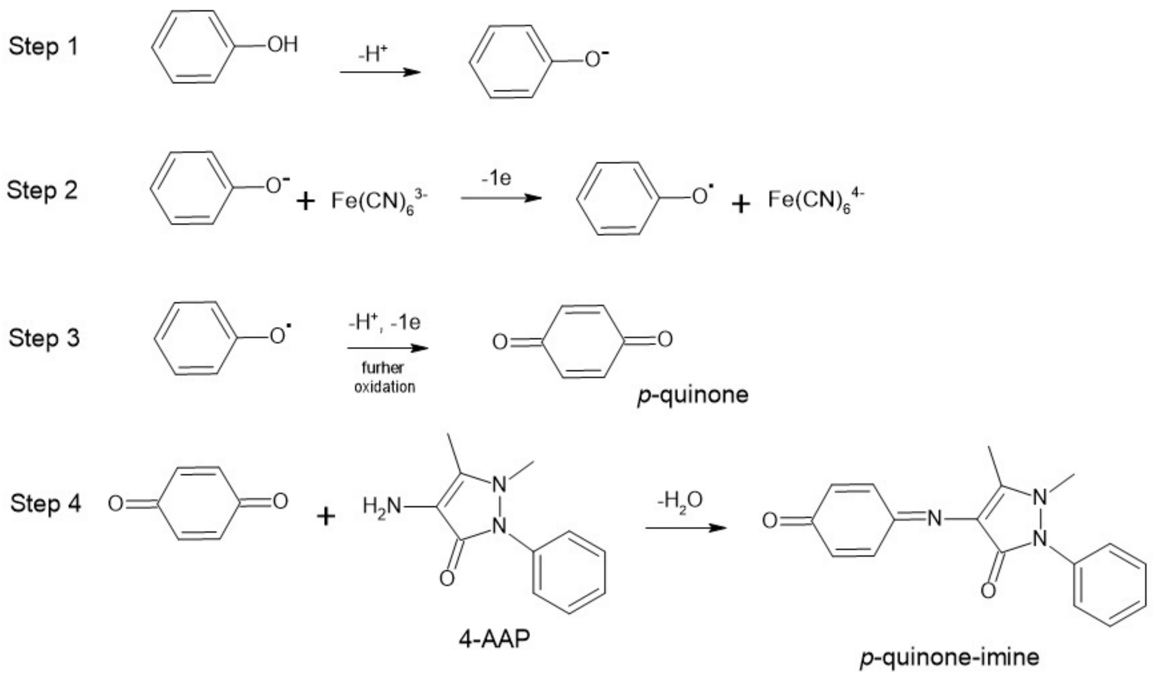

Scheme S1. Proposed mechanism for molecules containing only one phenolic group using Emerson's reagent.

Proposed mechanism:

1. Step 1: Under alkaline conditions, phenoxide ions are formed.
2. Step 2: Single-electron oxidation by $Fe(CN)_6^{3-}$ generates phenoxy radicals and $Fe(CN)_6^{4-}$.
3. Step 3: Further oxidation leads to the formation of *para*-quinones, albeit in low yield.
4. Step 4: In the presence of 4-aminoantipyrine (4-AAP – solution D), this compound acts as a nucleophile and couples with the oxidised phenol – typically at the *para*-position – forming a highly conjugated quinone-imine derivative on the pyrazole ring, responsible for the intense red/orange colouration.

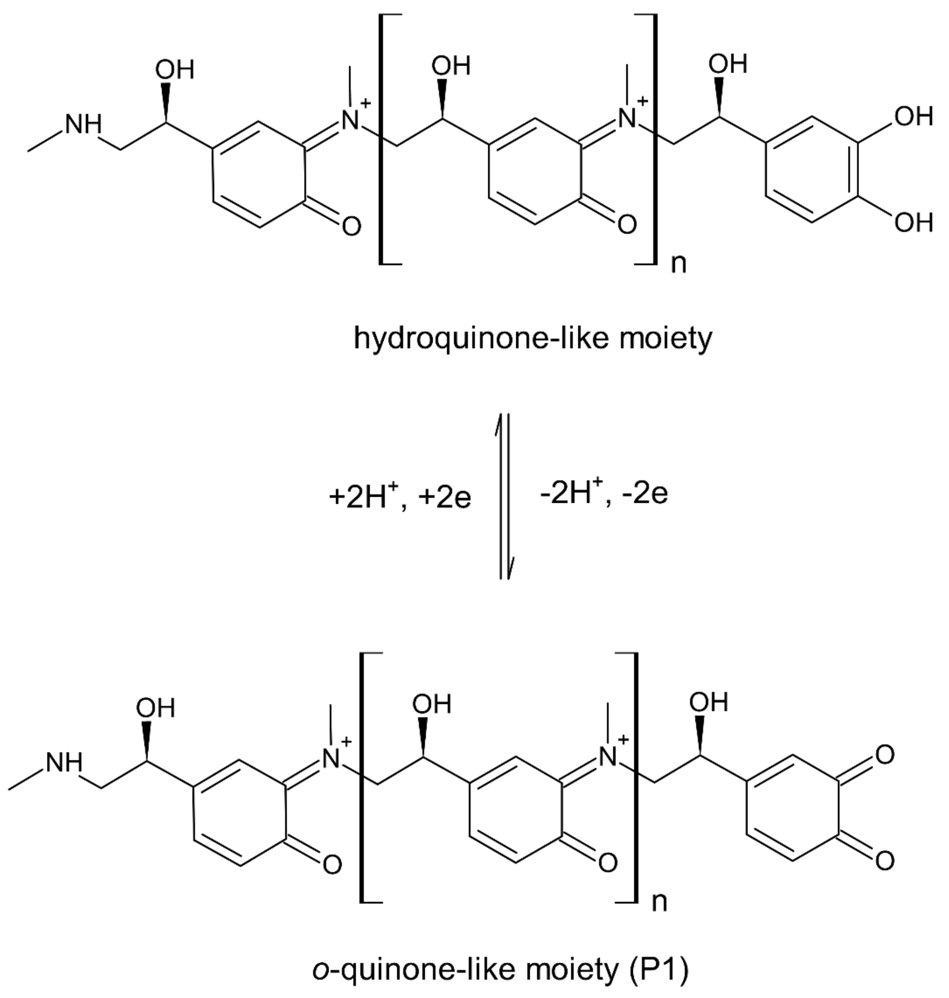

Scheme S2. Proposed mechanism for electrochemical redox processes after positive CR.

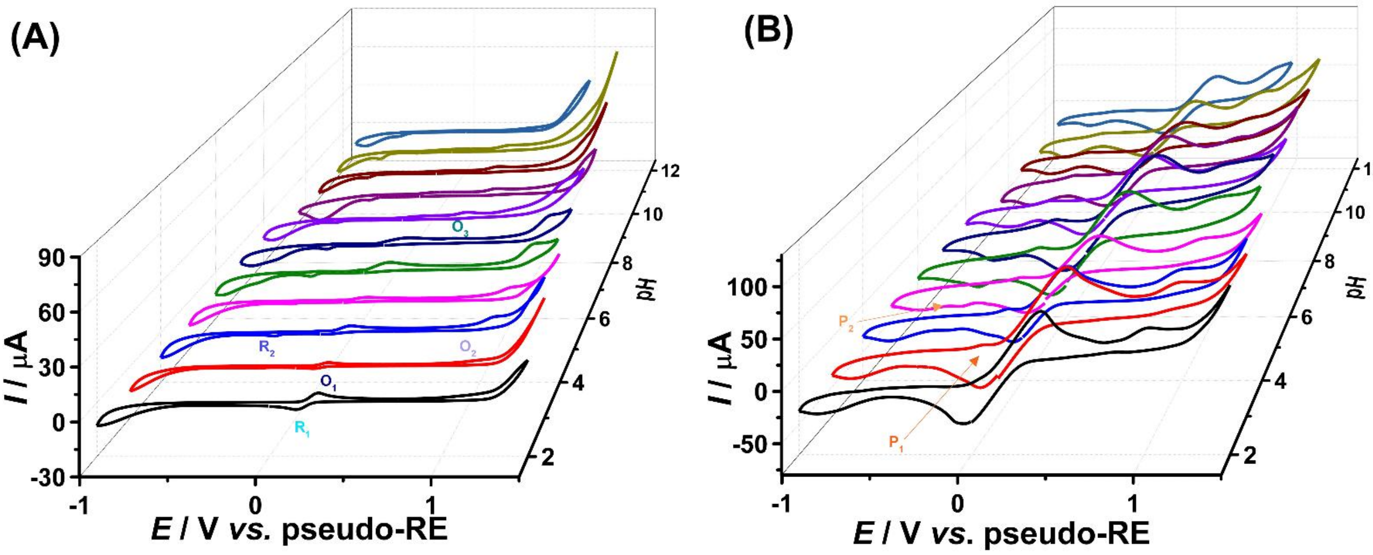

Figure S1. 3D plots of recorded cyclic voltammograms (first scan) of 66.0 μg L^{-1} EPN in 0.1 mol L^{-1} BR buffer solution with different pH values (from 2 to 12), before (**A**) and after the

CR by Melo Test (**B**) on SPE-Gr. All potential scans started at 0.0 V (*vs*. pseudo-RE), with a scan rate of 50 mV s^{-1}.

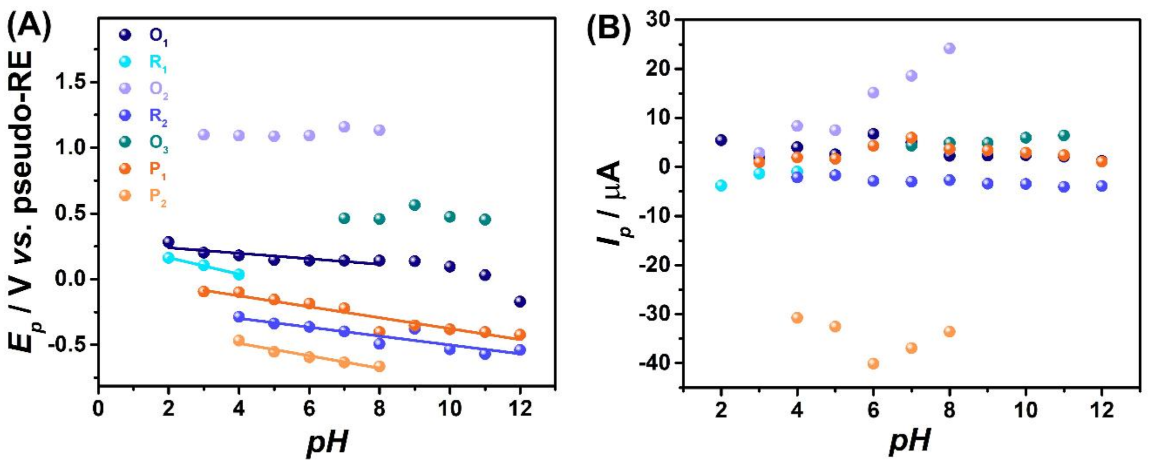

Figure S2. Plot of E_p (**A**) and I_p (**B**) *vs*. pH for the EPN redox processes on SPE-Gr before (**O_1**, O_2, **O_3**, **R_1** and **R_2**) and after CR (**P_1** and **P_2**), obtained by the recorded voltammograms shown in Figure S1.

Table S1. Linear relationship between E_p and pH for EPN processes

	Redox process	pH range	Linear regression equation	R^2
	**O_1**	2 – 8	E_p (V) = 0.28 (±0.03) – 0.021 (±0.006) × *pH*	0.86
**Before CR**	**R_1**	2 – 4	E_p (V) = 0.29 (±0.01) – 0.063 (±0.004) × *pH*	0.99
	**R_2**	4 – 12	E_p (V) = – 0.16 (±0.05) – 0.033 (±0.006) × *pH*	0.90
**After CR**	**P_1**	3 – 12	E_p (V) = 0.04 (±0.04) – 0.042 (±0.005) × *pH*	0.94
	**P_2**	4 – 8	E_p (V) = –0.29 (±0.03) – 0.047 (±0.005) × *pH*	0.97

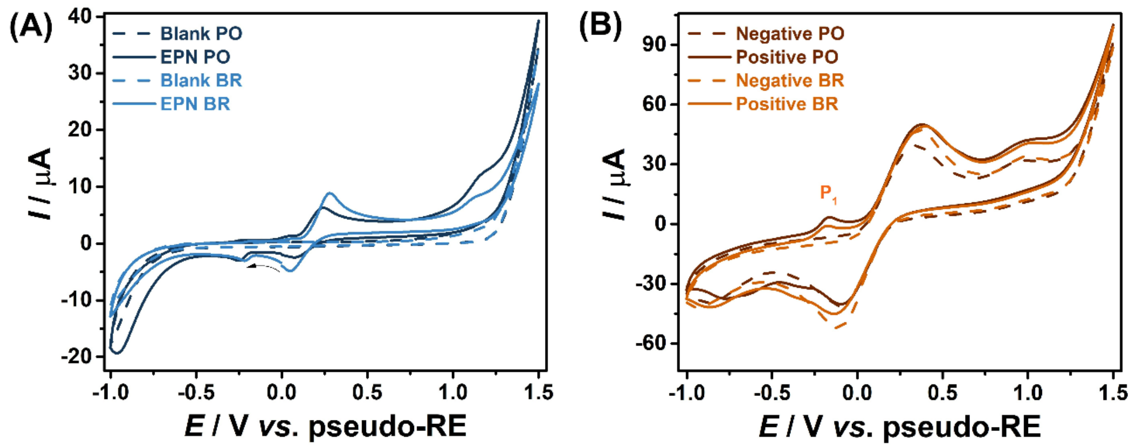

Figure S3. Cyclic voltammograms of 66.0 µg L^{-1} EPN on a SPE-Gr in 0.1 mol L^{-1} BR (blue light and orange light lines) and phosphate (dark blue and dark orange) buffer solutions (pH 3) - solid lines - and blank solutions (dashed lines) recorded before (**A**) and after (**B**) the CR by Melo Test. All potential scans started at 0 V, with a scan rate of 50 mV s^{-1}.

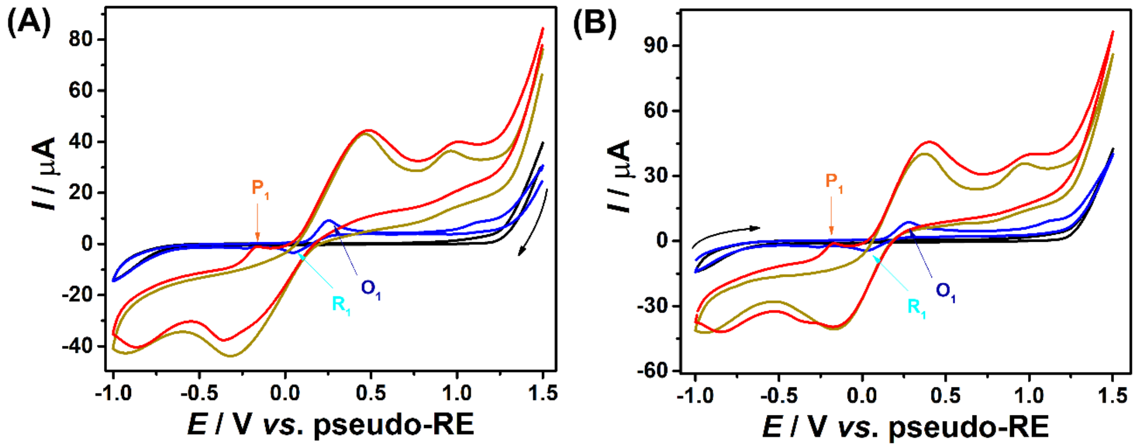

Figure S4. Cyclic voltammograms of 0.1 mol L^{-1} BR buffer solution pH 3 before (black line) and after addition of 66.0 µg L^{-1} EPN (blue line); and the negative control of CR (yellow line) and after CR by Melo Test with presence of EPN (red line) – for cathodic (**A**) and anodic (**B**) scans on a SPE-Gr. All potential scans started at 0 V, with a scan rate of 50 mV s^{-1}.

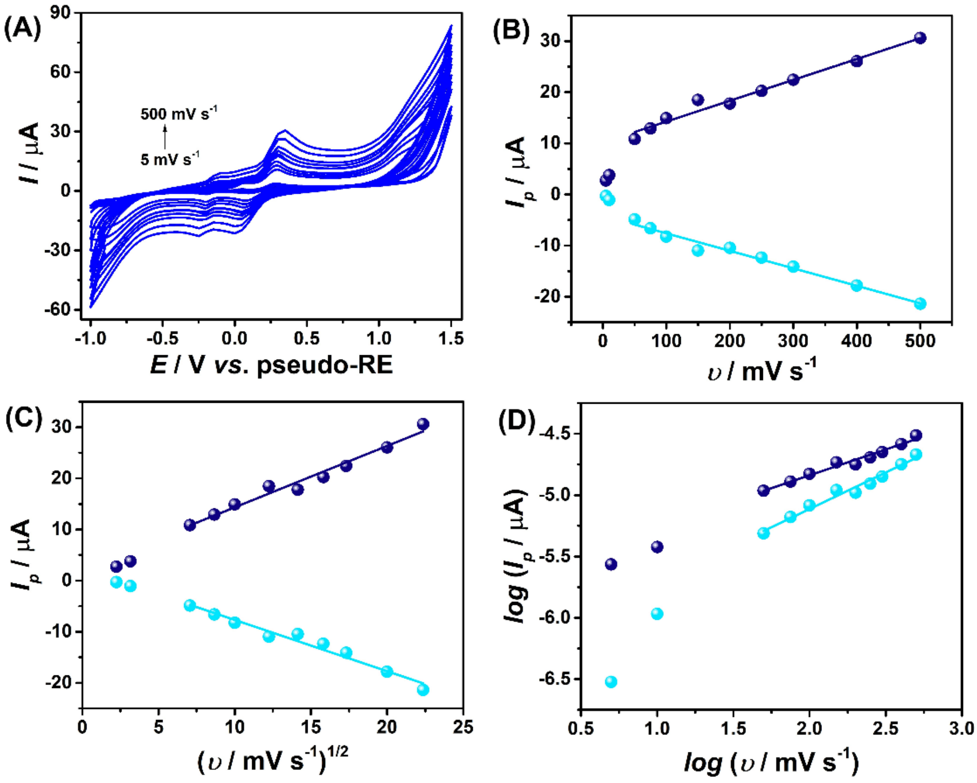

Figure S5. (**A**) Cyclic voltammograms at scan rates ranging from 5 to 500 mV s^{-1} on SPE-Gr for 66.0 µg L^{-1} EPN in 0.1 mol L^{-1} BR buffer solution at pH 3 (before CR). Plots of the linear regressions obtained for (**B**) I_p *vs.* v; (**C**) I_p *vs.* v^2, and (**D**) log I_p *vs.* log v are also shown. In dark blue **O_1** and in light blue R_1.

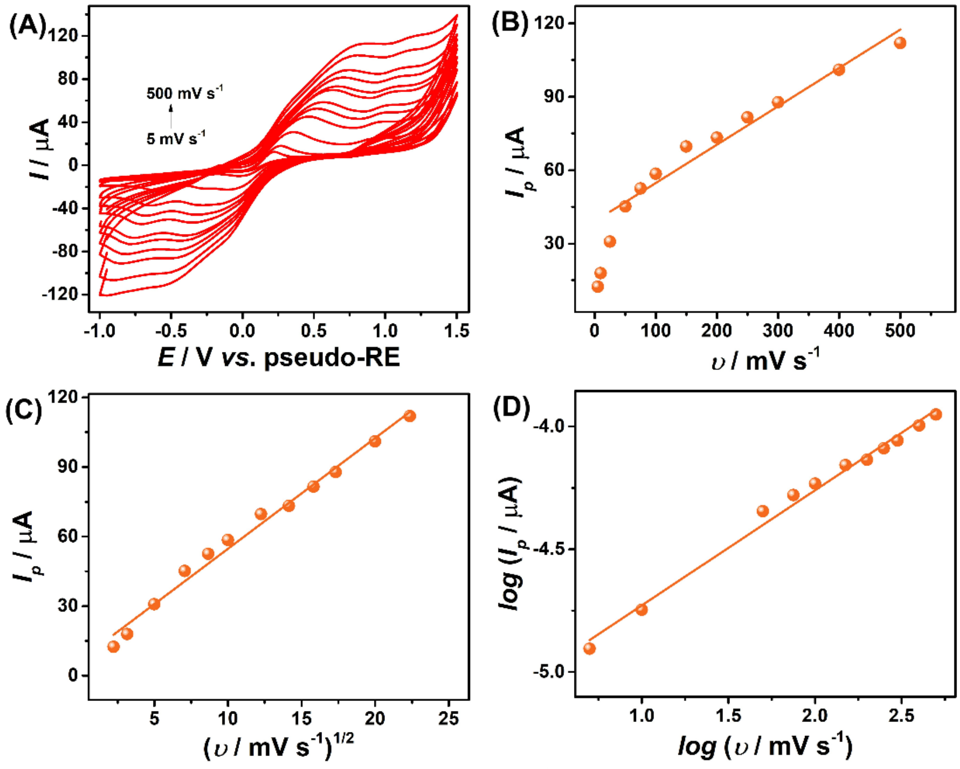

Figure S6. (**A**) Cyclic voltammograms at scan rates ranging from 5 to 500 mV s^{-1} on SPE-Gr for 66.0 µg L^{-1} EPN in 0.1 mol L^{-1} BR buffer solution at pH 3 (after CR). Plots of the linear

regressions obtained for (**B**) I_p *vs.* v; (**C**) I_p *vs.* v^2, and (**D**) log I_p *vs.* log v are also shown. In dark orange **P_1**.

Table S2. Linear regression equations and R^2 values of the plots of log I_p *vs.* log v

	Redox process	Linear regression equation	R^2
Before CR	**O_1**	$log\ I_p$ (μA) = – 5.68 (± 0.06) + 0.42 (± 0.03) × $log\ v$ (mV s^{-1})	0.98
	**R_1**	$log\ I_p$ (μA) = – 6.30 (± 0.08) + 0.50 (± 0.04) × $log\ v$ (mV s^{-1})	0.98
After CR	**P_1**	$log\ I_p$ (μA) = – 5.19 (± 0.03) + 0.47 (± 0.02) × $log\ v$ (mV s^{-1})	0.99

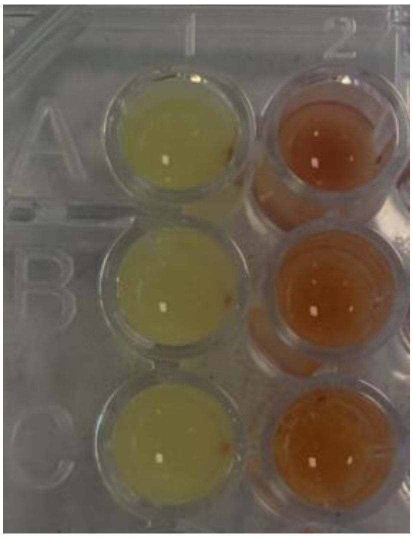

Figure S7. Result of the Melo Test in the absence (left) and presence (right) of 1 mg L^{-1} EPN.

Table S3. Linear regression equations and R^2 values of the plots of I_p *vs.* [*EPN*]

	Redox process	Linear range / μg L^{-1}	Y - Intercept	Slope	R^2
Before CR	**O_1**	6 – 60	1.97 (±0.71)	0.38 (±0.02)	0.990
After CR	**P_1**	26.6 – 60	–11.7 (±1.9)	0.57 (±0.04)	0.989

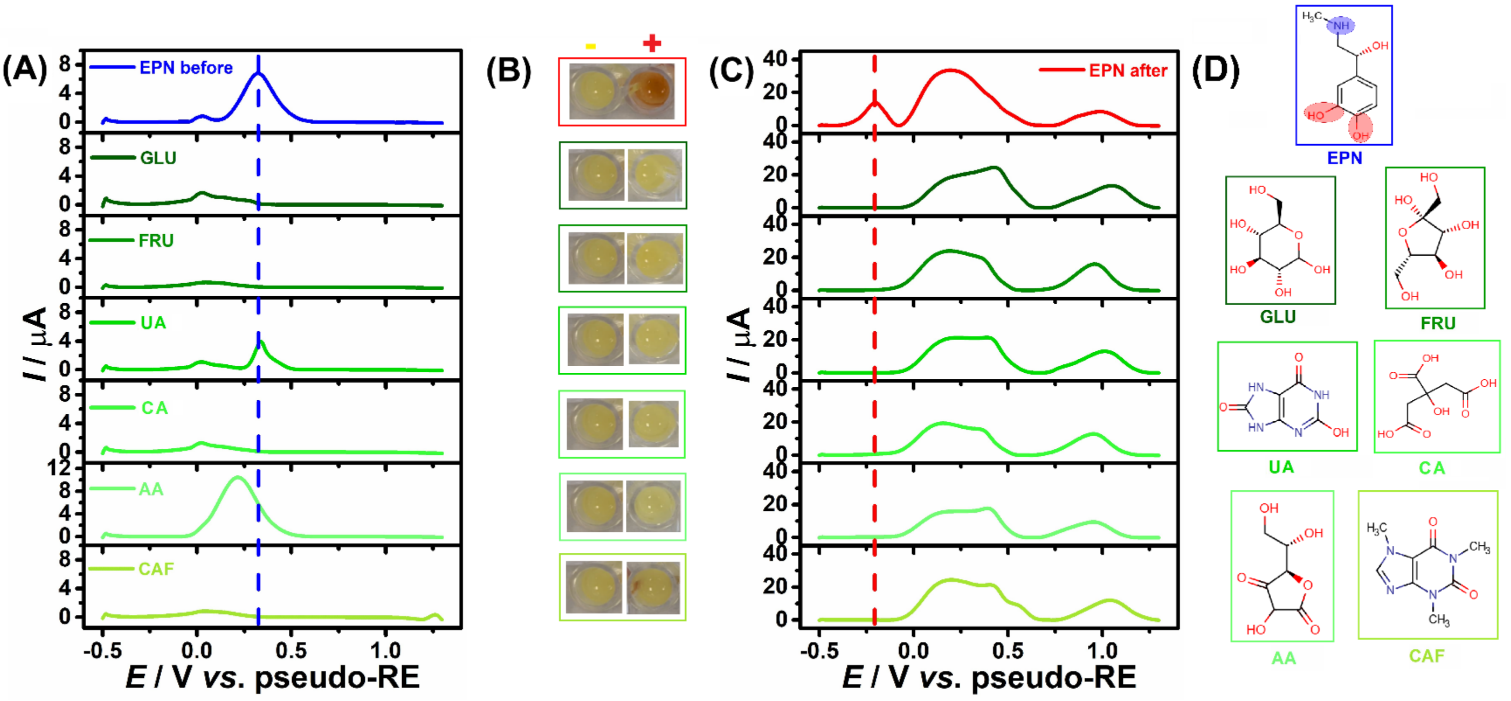

Figure S8. Square-wave voltammograms before (**A**) and after (**C**) CR on SPE-Gr. EPN before (blue line) after (red line) Melo Test. All compounds were at 30.0 µg L^{-1} in 0.1 mol L^{-1} BR buffer solution at pH 3. The experimental conditions were the same as in Fig.3. (**B**) Results of the Melo Test: (–) negative control and (+) EPN, GLU, FRU, UA, CA, AA and CAF, all at 0.6 mg mL^{-1}. (**D**) Chemical structure of all analytes in the same colours as (A).

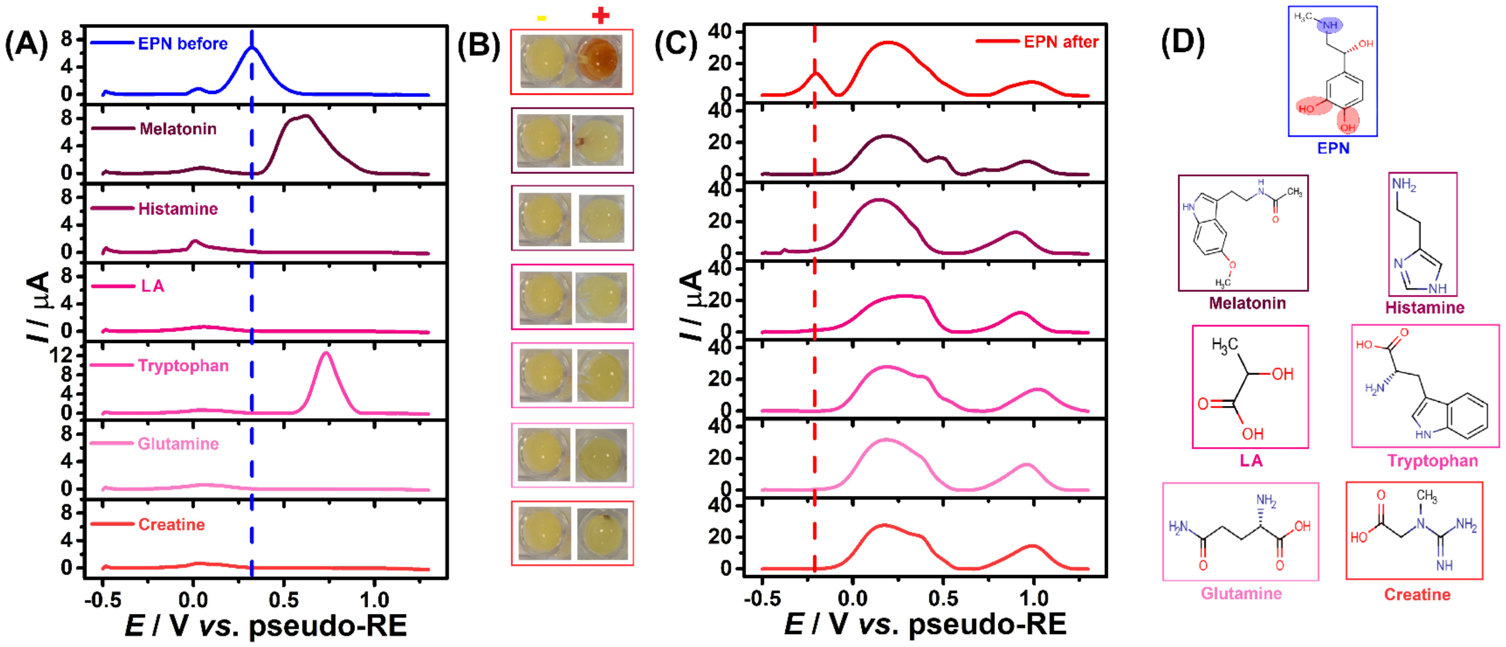

Figure S9. Square-wave voltammograms before (**A**) and after (**C**) CR on SPE-Gr. EPN before (blue line) and after (red line) Melo Test. All compounds were at 30.0 µg L^{-1} in 0.1 mol L^{-1} BR buffer solution at pH 3. The experimental conditions were the same as in Fig.3. (**B**) Results of the Melo Test: (–) negative control and (+) EPN, Melatonin, Histamine, LA, Tryptophan, Glutamine and Creatinine, all at 0.6 mg mL^{-1}. (**D**) Chemical structure of all analytes in the same colours as (A).

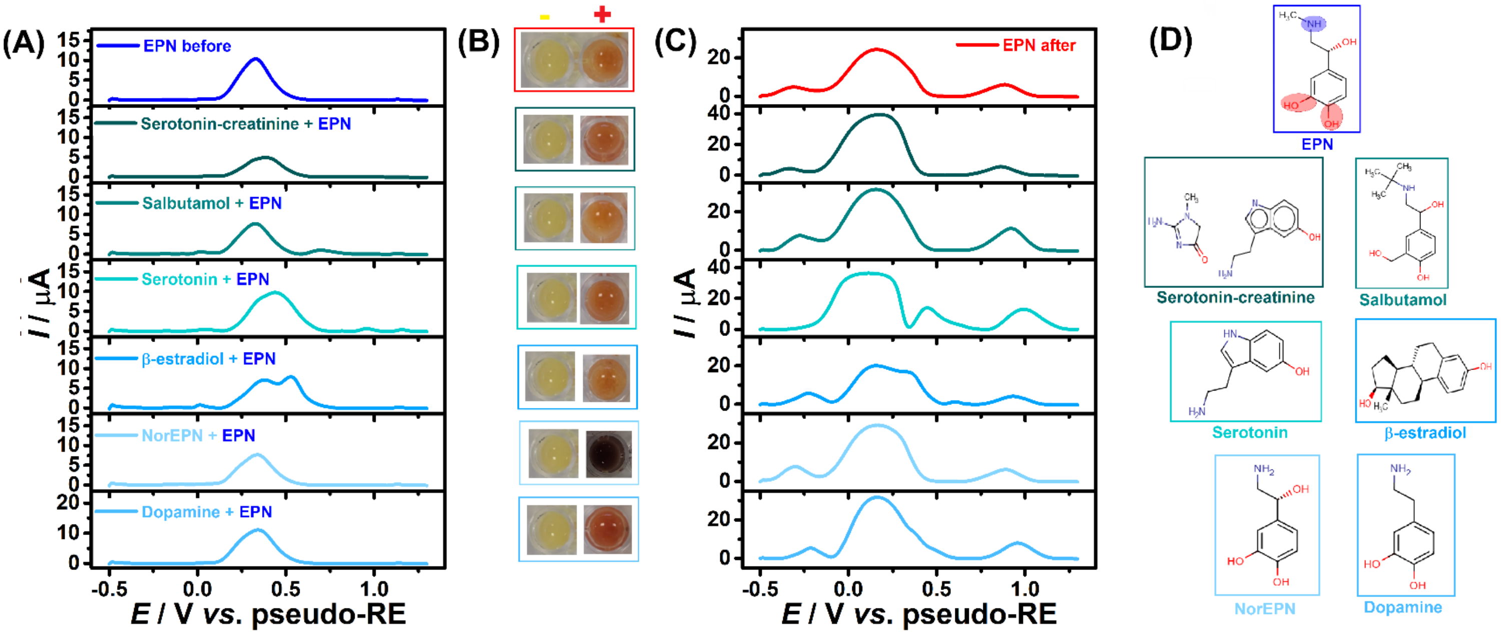

Figure S101. Square wave voltammograms before (**A**) and after (**C**) CR on SPE-Gr. EPN before (blue line) and after (red line) Melo Test. EPN at 20.0 µg L^{-1} and all other compounds at 10.0 µg L^{-1} in 0.1 mol L^{-1} BR buffer solution at pH 3. The experimental conditions were the same as in Fig.3. (**B**) Results of the Melo Test: (–) negative control and (+) EPN, Serotonin-creatinine, Salbutamol, Serotonin, β-estradiol, nor-EPN and Dopamine in mixture (2:1) with EPN (0.6 mg mL^{-1}). (**D**) Chemical structure of all analytes in the same colours as (**A**).

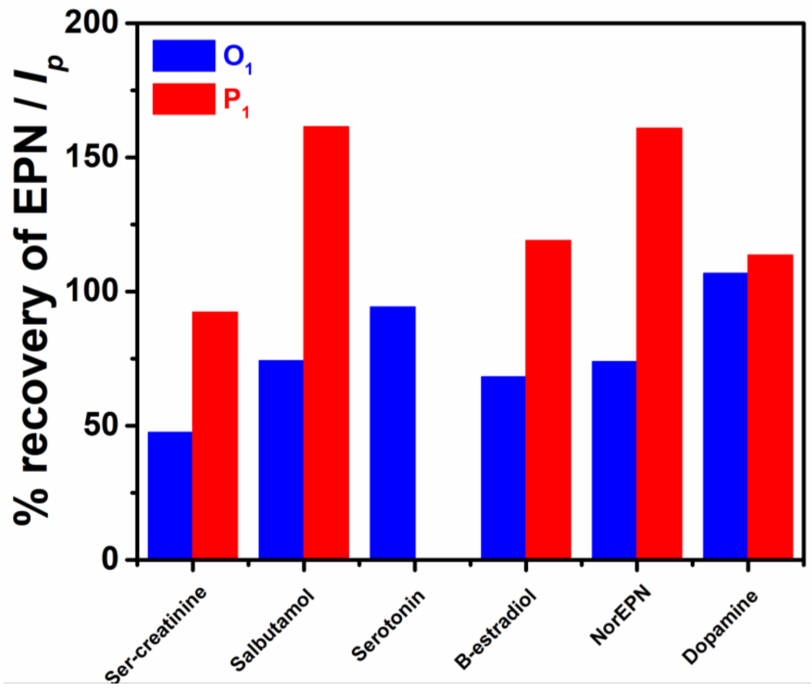

Figure S11. Recovery results obtained for **O_1** in blue and **P_1** in red, in solutions containing simultaneously EPN and each of the potential interferents (serotonin-creatinine, salbutamol, serotonin, β-estradiol, nor-EPN, and dopamine).

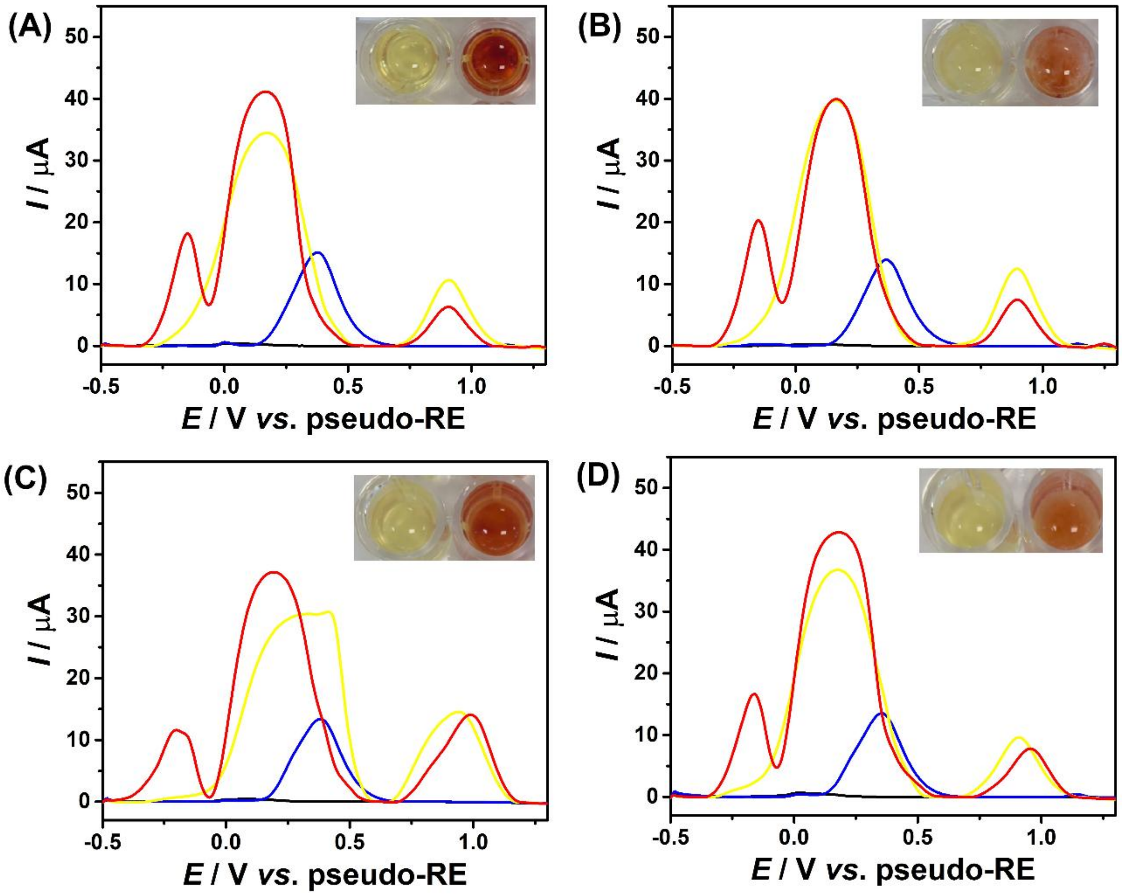

Figure S12. Square-wave voltammograms in 0.1 mol L^{-1} BR buffer solution (pH 3) on SPE-Gr before (black line) and after (blue line) addition of 30 µg L^{-1} EPN – before CR, and after CR: the negative control (dark yellow line) and the positive by Melo Test for presence of EPN (red line) in samples: vitreous humour (**A**), saliva (**B**), serum (**C**) and urine (**D**). Inset results of the Melo Test for negative control (yellow) and solutions in concentrations 0.6 mg mL^{-1} EPN. The experimental conditions were the same as in Figure 3.
